# Supplementary material for: Psychometric Qualities Evaluation of the Interdependent Happiness Scale across Malaysia, Philippines, and India
Source: Int J Environ Res Public Health. 2021 Dec 24;19(1):187. doi: 10.3390/ijerph19010187 (PMC8751005; doi:10.3390/ijerph19010187)
Supplement: Supplementary file 1 [file ijerph-19-00187-s001.zip › ijerph-1467285-supplementary.pdf]

Supplementary Table S1

Factor loading, descriptive statistics, and reliability of the Interdependent Happiness Scale with 8 items

Item/Factor	Malaysia (*n* = 263)					India (*n* = 310)				
	FL	*M*	*SD*	α	ω	FL	*M*	*SD*	α	ω
Relationship-oriented happiness	.770	3.72	0.71	.688	.693	.941	3.90	0.77	.853	.854
IHS2	.759	3.60	0.85			.846	3.85	0.86		
IHS3	.694	3.83	0.78			.878	3.94	0.80		
Quiescent happiness	.896	3.47	0.76	.652	.642	1.00	3.79	0.78	.792	.844
IHS4	.643	3.90	0.85			.798	3.94	0.81		
IHS5	.586	3.00	1.11			.759	3.57	1.10		
IHS6	.634	3.50	1.02			.791	3.86	0.85		
Ordinary happiness	.939	3.35	0.83	.795	.705	.925	3.72	0.84	.900	.895
IHS7	.708	3.28	1.00			.862	3.71	0.91		
IHS8	.634	3.22	1.01			.880	3.71	0.89		
IHS9	.785	3.54	0.94			.853	3.74	0.96		
8-item IHS	-	3.49	0.64	.830	.840	-	3.79	0.74	.931	.956

Note. FL = standardized factor loading, M = mean, SD = standard deviation, α = Cronbach alpha coefficient, ω = McDonald omega coefficient, IHS: overall interdependent happiness scale score with 8 items.

Supplementary Table S2

Correlation, squared correlation, and average variance extracted of the Interdependent Happiness Scale with 8 items

	1	2	3	4	5	AVE
**Malaysia**						
1.IHS	1	.513	.719	.764	.080	.457
2.Relationship	.716***	1	.205	.239	.106	.532
3.Quiescent	.848***	.453***	1	.325	.044	.381
4.Ordinary	.874***	.489***	.570***	1	.043	.501
5.Creativity	.284***	.325***	.209**	.208**	1	-
**India**						
1.IHS	1	.748	.903	.880	.404	.689
2.Relationship	.865***	1	.591	.498	.441	.741
3.Quiescent	.950***	.769***	1	.692	.329	.605
4.Ordinary	.938***	.706***	.832***	1	.308	.748
5.Creativity	.636***	.664***	.574***	.555***	1	-

Note. AVE: average variance extracted; IHS: overall interdependent happiness scale score with 8 items; Relationship: relationship-oriented happiness score with items 2 and 3. Above diagonal line is the squared correlation coefficient.

** $p < .01$, *** $p < .001$
